# Hydrogen Storage in Bilayer Hexagonal Boron Nitride: A First-Principles Study

**DOI:** 10.1021/acsomega.1c03443

**Published:** 2021-11-03

**Authors:** Dibya Prakash Rai, Bhanu Chettri, Prasanta Kumar Patra, Shahid Sattar

**Affiliations:** † Physical Sciences Research Center (PSRC), Department of Physics, Pachhunga University College, Mizoram University, Aizawl 796001, India; ‡ Department of Physics, North-Eastern Hill University, Shillong 793022, Meghalaya, India; § Physical Sciences Research Center (PSRC), Department of Physics, Pachhunga University College, Aizawl 796001, Mizoram, India; ∥ Department of Physics and Electrical Engineering, Linnaeus University, Kalmar SE-39231, Sweden

## Abstract

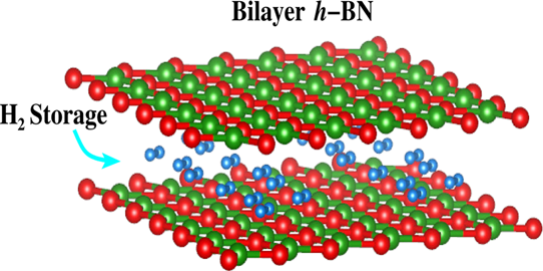

Using first-principles calculations, we report on the structural and electronic properties of bilayer hexagonal boron nitride (*h*-BN), incorporating hydrogen (H_2_) molecules inside the cavity for potential H_2_-storage applications. Decrease in binding energies and desorption temperatures with an accompanying increase in the weight percentage (upto 4%) by increasing the H_2_ molecular concentration hints at the potential applicability of this study. Moreover, we highlight the role of different density functionals in understanding the decreasing energy gaps and effective carrier masses and the underlying phenomenon for molecular adsorption. Furthermore, energy barriers involving H_2_ diffusion across minimum-energy sites are also discussed. Our findings provide significant insights into the potential of using bilayer *h*-BN in hydrogen-based energy-storage applications.

## Introduction

Rising energy demands due to rapid increase in population density and depletion of natural resources is a matter of grave concern and a major threat to a sustainable future. Carbon emissions owing to the excessive burning of fossil fuels have resulted in unprecedented environmental changes in recent times, which have drastically impacted all living creatures on this planet. To tackle this, an effective and efficient approach to balance industrial developments alongside preserving natural deposits for a sustainable life is highly desirable. Therefore, enormous efforts are ongoing to capture CO_2_ discharge for the purification of air,^1−3^ though it is also challenging and costly. While the most effective way of capturing CO_2_ is fast afforestation which is hard to accomplish due to the limitations of land and rampant deforestation, use of cleaner energy resources as an alternative in factories and vehicles could partly solve issues. In this regard, proposals are underway using hydrogen (H_2_) as an alternative fuel, a highly combustible gas promising for automobiles in the form of fuel cells, to tackle the global energy crisis with a minimal impact on the environment.^4−8^

Hydrogen is generated from biomass and via steam reforming of natural gas such as methane and water electrolysis which falls under fossil fuels and renewable resources.^9^ Methods such as photobiological and photochemical water splitting are under development, whereas processes such as alkaline water electrolysis, solid oxide electrolysis, and proton exchange membrane (PEM) water electrolysis are some of the established hydrogen production methods.^10^ PEM water electrolysis which contributes to around 4% of the global hydrogen production has been considered one of the most prominent techniques to generate clean and efficient hydrogen with a high production rate from renewable energy sources without any pollutants as byproducts. Moreover, biophotolysis, steam reforming, autothermal reforming, electrolysis, dark fermentation, and so forth have been proven to have an efficiency of above 40% but lack in prospects of storage, high capital costs, transportation, and clean byproducts. Around 70 million tons per annum of hydrogen are produced around the world of which a large fraction is utilized for industrial purposes.^11,12^ Because oxygen (O_2_) supports the combustion (2H_2_+O_2_ → 2H_2_O+Δ*E*), the energy derived out of burning H_2_ leads to water (H_2_O) as a natural byproduct and favors its utilization as a fuel in reducing the effects of CO_2_ emissions. Foreseeing the aforementioned prospects, a technical approach to discover potential new materials for efficient reversible H_2_ storage is much needed.^13,14^

The discovery of graphene opened doors to a new era of technological revolution.^15−20^ Interest in two-dimensional (2D) nanomaterials thereafter has grown exponentially finding applications in nanoscale digitization, spintronics, gas sensing, catalysis, and H_2_ production and storage, to list a few.^21−30^ Due to their largest surface area-to-volume ratio and exceptional chemical stability, graphene-like materials^31,32^ such as graphdiyne,^33,34^ honeycomb BC_3_,^35,36^ borophene,^37−43^ CN,^44,45^ and g-C_3_N_4_^46−49^ layer have already been investigated and recognized as a safe reservoir for H_2_ storage. Moreover, the search of potential H_2_-storage materials in other 2D layered structures such as hexagonal boron nitride (*h*-BN),^28,50^ boron arsenide,^51^ black and blue phosphorene,^51−57^ boron monochalcogenides,^58^ and gallium monochalcogenides^59−62^ is still ongoing.

H_2_ gas is mostly preserved either by liquefaction under high compressing pressure^63−66^ or by adsorption on the surface or interstitial region of material cavity.^67−71^ In relation to this, the adsorption of H_2_ on the surface of 2D materials has advantages in terms of safe functionality and cost-effectiveness. For the effective utilization of H_2_ in fuel cells, the adsorption energy and gravimetric weight percentage on the adsorbent should be sufficiently high.^72,73^ Recently, vehicles having H_2_ fuel cells with a gravimetric weight percentage of 6% were successfully tested.^74^ The adsorption/desorption kinetics and the strength of binding energy ought to be intermediate for hydrogen to bind on the material surfaces with an optimal adsorption energy range. Owing to the obvious reasons mentioned above and the survey of previous works,^75−81^ 2D bilayer *h*-BN looks promising with numerous adequate functional properties such as high mechanical stability, carrier mobility, and outstanding electronic and optical properties which encourages its further utilization for energy-storage applications.^82−86^ Motivated by this, we explore the possibility of using bilayer *h*-BN for H_2_ storage by employing first-principles calculations. We analyzed trends in binding energy, desorption temperature, effective mass of electron and holes, and subsequently their effects on the structural and electronic properties of bilayer *h*-BN using state-of-the-art computational techniques.

## Computational Details

**Figure 1:**
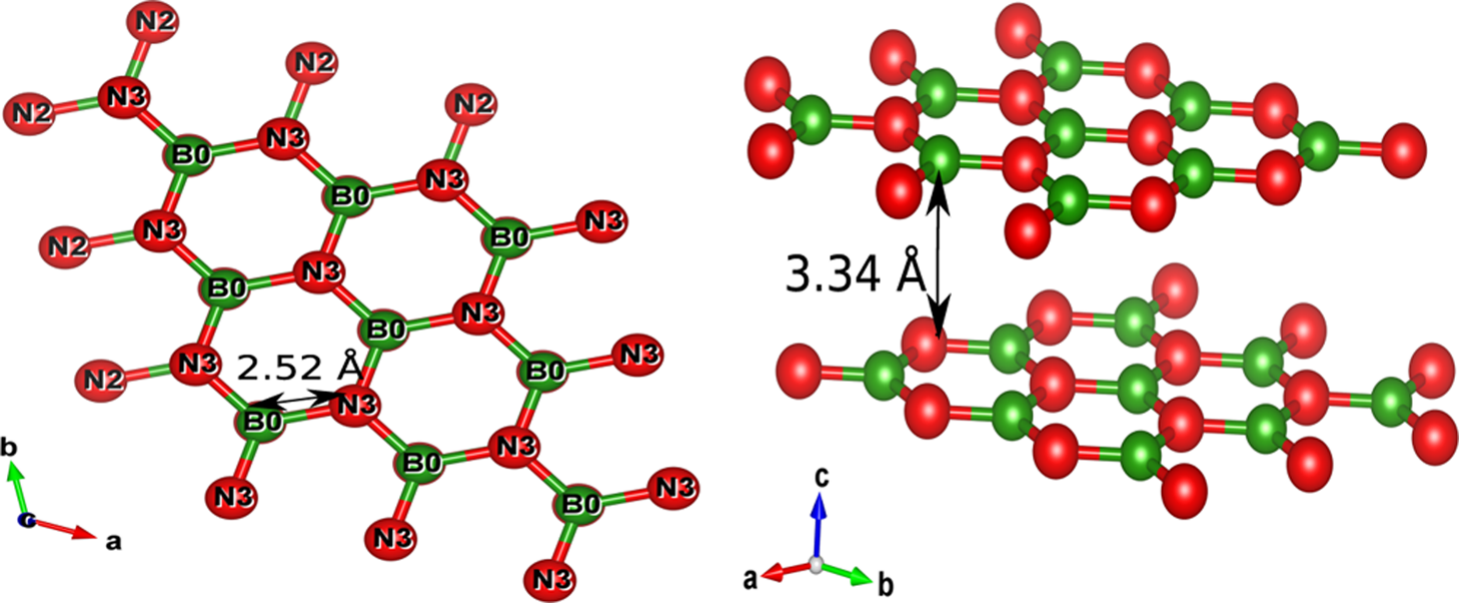
Top view and side view of pristine bilayer *h*-BN.

Bilayer *h*-BN was constructed by stacking two *h*-BN monolayers in a AA′-stacking configuration which has been proven to be the most stable configuration,^87,88,91^ having an in-plane lattice constant of *a* = 2.488 Å.^89,90^ A vacuum of 15 Å is inserted in the out-of-plane direction to avoid spurious interactions of wave functions. We used a 3 × 3 × 1 supercell consisting of 36 atoms with the B/N stoichiometric ratio of 1:1 as shown in Figure 1. The van der Waals interactions^91^ were incorporated using Grimme’s DFT-D2 scheme.^92^ The geometry of bilayer *h*-BN was optimized within the force field approximation using an interatomic potential developed by Stillinger Weber.^93,94^ Because bilayer *h*-BN can accommodate eight H_2_ molecules between the layers at its optimal capacity, geometrical relaxations were performed for all cases after inserting H_2_ molecules. Moreover, the electronic properties were computed at the level of generalized gradient approximations (GGAs) of type Perdew–Burke–Ernzerhof^95^ and DFT-1/2,^96,97^ and a comparison has been made between both approaches. As both GGA and DFT-1/2 were treated as semilocal functionals within the framework of Kohn–Sham density functional theory (DFT),^98^ self-consistency is achieved in the iterative solution for each case. A basis of the linear combination of atomic orbitals has been opted as programmed in the Quantumwise VNL-ATK package.^99^ Finally, a detailed study of each system was made possible while integrating the first Brillouin zone with dense Monkhorst–Pack 16 × 16 × 1 *k*-mesh,^100^ and the *k*-point convergence plot is supplied in the Supporting Information. Also, for the sake of reproducibility of our work, the relaxed atomic coordinates of the *h*-BN bilayer and the hydrogen-adsorbed system are provided in the Supporting Information.

## Results and Discussion

**Figure 2:**
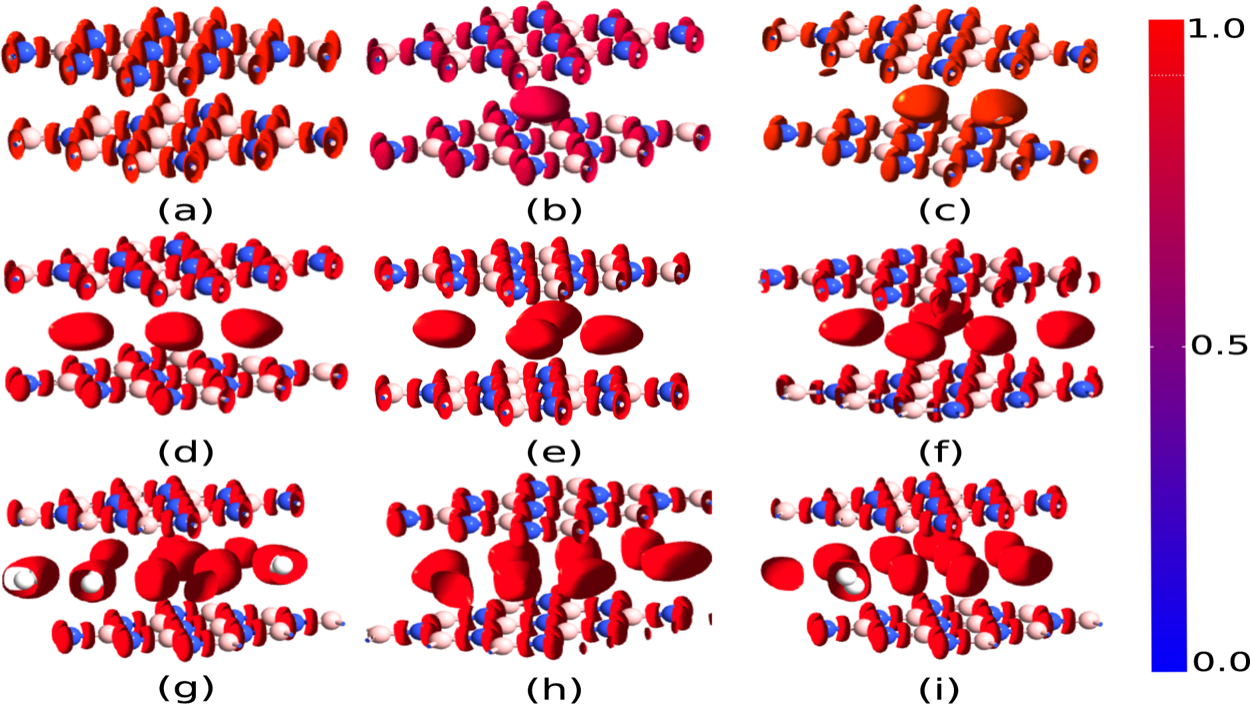
ELF of bilayer *h*-BN with the number of *H*_2_ molecules (a) *n*_H_ = 0, (b) *n*_H_ = 1, (c)*n*_H_ = 2, (d) *n*_H_ = 3, (e) *n*_H_ = 4, (f) *n*_H_ = 5, (g) *n*_H_ = 6, (h) *n*_H_ = 7, and (i) *n*_H_ = 8.

The optimized in-plane lattice parameters of 2.52 Å and the interlayer spacing of 3.34 Å for the pristine bilayer *h*-BN are in good agreement with previous studies.^89,90,101^ The ability of *h*-BN as a hydrogen-storage material relies on accommodating maximal number of H_2_ molecules while maintaining the stability (without undergoing structural deformation). In this regard, geometrical optimizations were performed by inserting H_2_ molecules one after the other in the cavity of bilayer *h*-BN. The hollow site (in the middle of the hexagonal crystals) turns out to be the minimum-energy configuration for which atomic relaxations were performed following the Broyden-Fletcher-Goldfarb-Shanno scheme by gradually increasing the H_2_ content. Figure 2a–i shows the electron localization function (ELF) of pristine and H_2_-adsorbed bilayer *h*-BN. The presence of electronic cloud between the B and N atoms shows the presence of intralayer covalent bonds, whereas bigger lobes around the H–H bond in between the cavity for each H_2_ molecule depicts the same.

**Figure 3:**
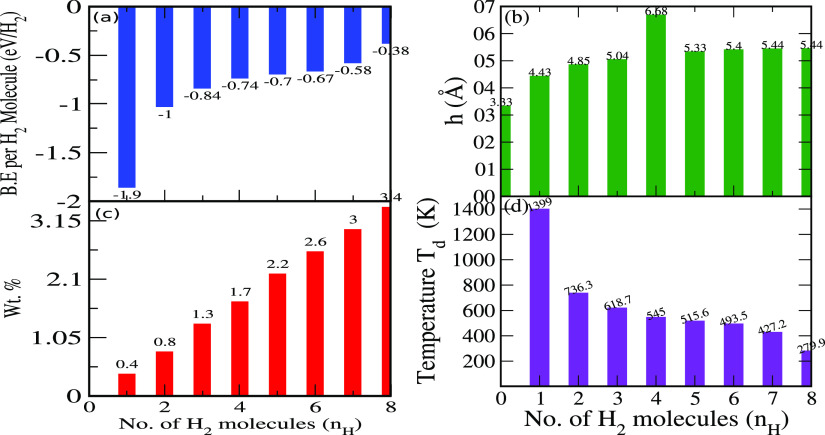
(a) Binding energy per H_2_ molecule. (b) Interlayer spacing between adjacent *h*-BN monolayers. (c) Wt % with respect to H_2_ content. (d) Desorption temperature (T_D_) of bilayer *h*-BN with the number of *H*_2_ molecules *n*_H_ = 0, 1, 2, 3, 4, 5, 6, 7, and 8.

We first check the stability of our systems by computing the binding energy (*E*_b_) per H_2_ molecule up to maximal H_2_ molecular capacity by using the following definition
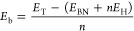
1where *E*_T_ is the total energy of the combined system (*h*-BN + H_2_), *E*_BN_ is the total energy of the pristine bilayer *h*-BN, *n* is the number of H_2_ molecules, and *E*_H_ refers to the total energy of H_2_ molecules. As shown in Figure 3a, *E*_b_ remains negative even for the optimal number of H_2_ molecules, which indicates stability of our systems in accommodating H_2_ molecules in the bilayer *h*-BN cavity. Moreover, the interlayer spacing between the neighboring *h*-BN layers was found to be increasing with the increase of H_2_ molecules (see Figure 3b). We attribute this to the expanded electronic cloud between the van der Waals gap of bilayer systems.

To provide a quantitative account of H_2_ storage, we also compute dimensionless weight percentage (Wt %) for all given cases in Figure 3c using the definition of eq 2(102)
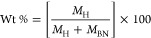
2where *M*_H_ and *M*_BN_ are the molecular masses of the H_2_ molecule and bilayer *h*-BN, respectively. As expected, the Wt % increases by adding more H_2_ molecular content (see Figure 3c). Because the bilayer cavity can hold up to eight H_2_ molecules, it gives a Wt % value of 4% which is slightly less than the previously reported value of 6%.^74^ Another key aspect in this realm is to analyze the reversibility kinematics (adsorption ⇌ desorption) for which we computed the desorption temperature (*T*_D_) for all H_2_ adsorbed bilayer systems using the Van’t Hoff equation^103,104^ given as
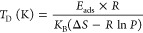
3where *E*_ads_ is the average adsorption energy, *R* = 8.3145 JK^–1^ Mol^–1^ is the gas constant, *K*_B_ = 1.38 × 10^–23^ JK^–1^ is Boltzmann constant, Δ*S* represents the change in H_2_ entropy from the gas to liquid phase, and *P* is the equilibrium pressure taken to be 1 atm, respectively. The calculated *T*_D_ ranges from 1399 K to 279 K when increasing the number of H_2_ molecules from one to eight, as shown in Figure 3d. Our results of *T*_D_ are above the room temperature (except for 8H_2_, which is just below the room temperature) up to the maximum gravimetric Wt % of 3.4%, indicating bilayer *h*-BN to be a potential material for storing H_2_ molecules at elevated temperatures.

**Figure 4:**
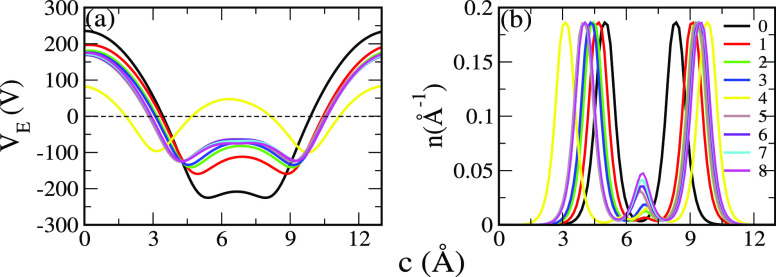
(a) Electrostatic potential (*V*_E_) and (b) charge density (*n*_E_) of bilayer *h*-BN with the number of H_2_ molecules *n*_H_ = 0, 1, 2, 3, 4, 5, 6, 7, and 8.

We next computed one-dimensional electrostatic potential (*V*_E_) and distribution of charge densities (*n*_E_) in the out-of-plane direction presented in Figure 4a,b. Referring to the black line in Figure 4a, it is noteworthy that the pristine bilayer has a flat shallow potential in between the two *h*-BN monolayers. However, by inserting H_2_ molecules in between the bilayer cavity, the shallow peak gradually rises analogous to the case of trap electrons in a typical square well potential. The peak intensity also increases with the increase of H_2_ molecular concentration, except for the case of *n* = 4 (yellow line in Figure 4a). Here, the potential peak crosses the Fermi level (*E*_F_) giving rise to the tunneling barrier effect. The tunneling barrier (Φ) measures the efficiency of the rate of charge transfer. In the case of 4H_2_, the stacking configuration is transformed from AA′- stacking configuration to AB stacking. Also, one of the H_2_ molecules under structural optimization moved in the vertical direction leading to an increase in the interlayer distance to 6.8 Å. The change in the stacking configuration and the movement of a single H_2_ molecule close to one of the layers have led to the change in the charge distribution, thus leading to the exceptional case for *n* = 4. Higher value of Φ is a hindrance to the charge transfer from *h*-BN → H_2_ molecules. Following the Bader charge analysis, this can be inferred by the increase in charge density at the hydrogen site by a small amount of 0.02e, 0.017e, 0.034e, 0.021e, 0.048e, 0.055e, 0.072e, and 0.079e for *n*_H_ = 0, 1, 2, 3, 4, 5, 6, 7, and 8, respectively. Because none of the potential peaks crosses *E*_F_ except at *n*_H_ = 4, we assume Φ = 0 for all systems which allows charge transfer near the Fermi level. We can also draw similar conclusions from the one-dimensional electron density (*n*) plot as shown in Figure 4b. Here, the peaks in the center are due to the accumulation of charge localization of H_2_ molecules whose intensity increases by the addition of H_2_ molecules. On the other side, no significant changes are observed in the charge density profiles at the B and N atomic sites. However, we notice that separation between the two peaks increases with the increase of H_2_ molecular content. We thus conclude that the increase in the number of H_2_ molecules creates a repulsive force between the two *h*-BN layers.

**Figure 5:**
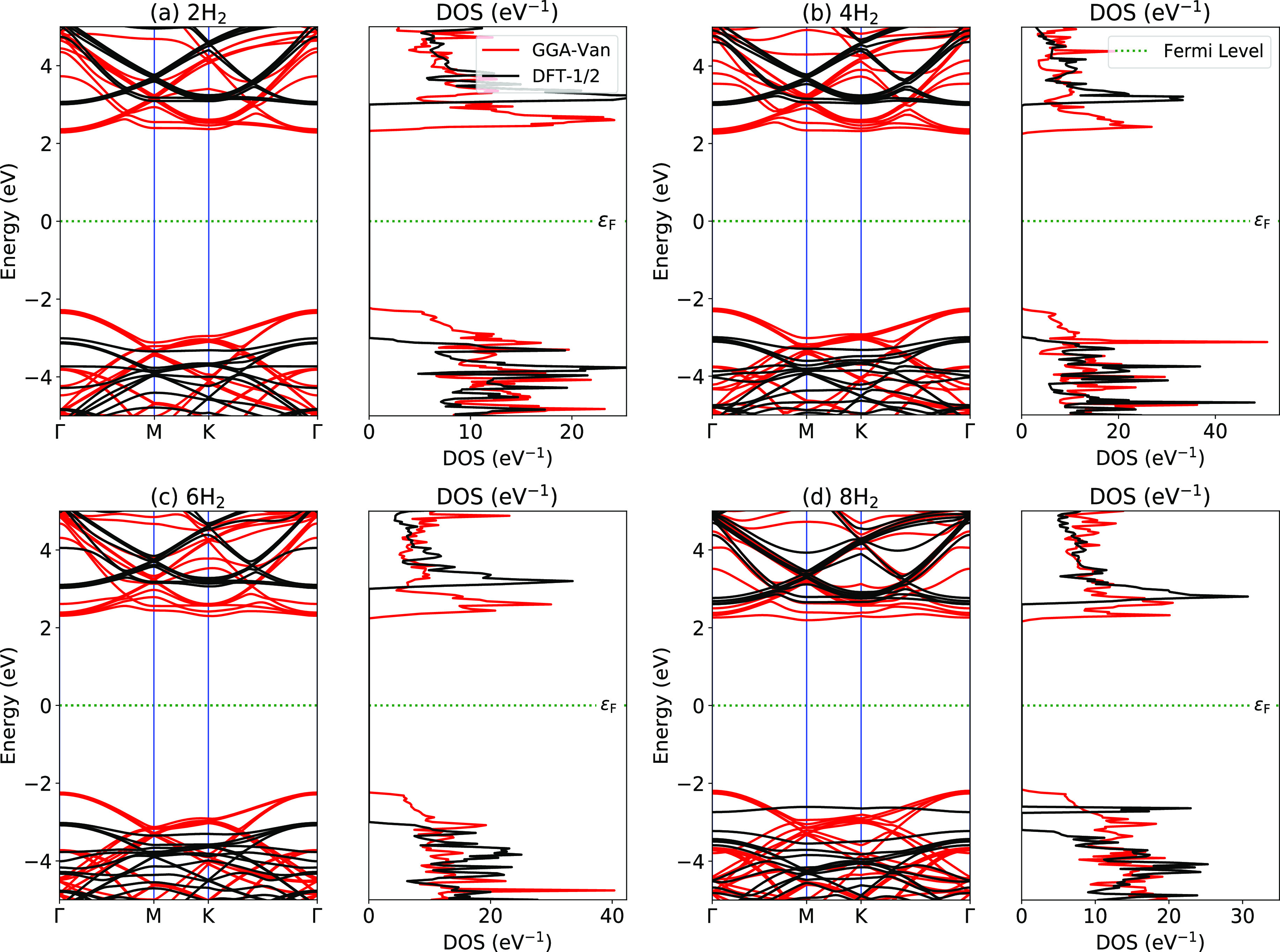
Electronic band structures and DOS of bilayer *h*-BN with number of H_2_ molecules (a) *n*_H_ = 2, (b) *n*_H_ = 4, (c) *n*_H_ = 6, and (d) *n*_H_ = 8. The rest of electronic band structures and DOS are provided in the Supporting Information.

**Figure 6:**
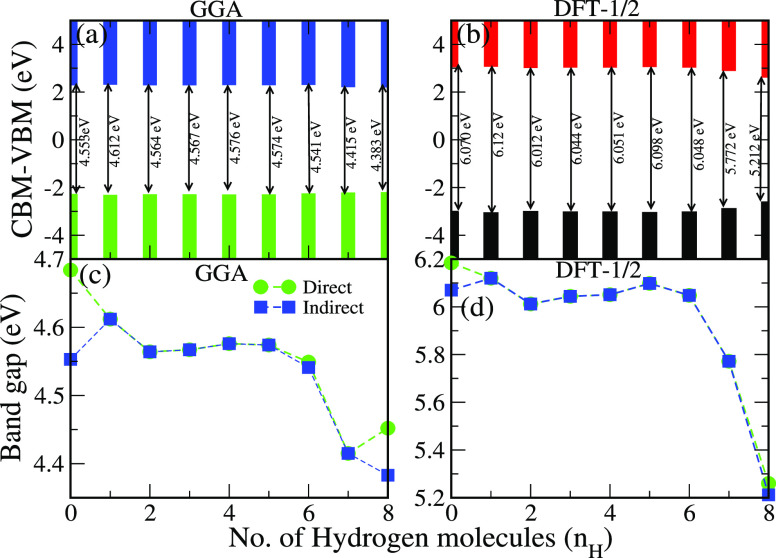
(a) Direct band gap using the GGA functional, (b) direct band gap using the DFT-1/2 functional, (c) direct–indirect band gap using GGA, and (d) direct–indirect band gap using DFT-1/2 of bilayer *h*-BN with the number of *H*_2_ molecules *n*_H_ = 0, 1, 2, 3, 4, 5, 6, 7, and 8.

We next examine the electronic behavior of all cases (H_2_ inserted bilayer *h*-BN) by calculating the electronic band structures and density of states (DOS) shown in Figure 5. In our previous work, we have already reported the accuracy of DFT-1/2 over GGA functionals in which an increase in band gap of *h*-ZnSe depicts consistency of results corresponding to other higher-order functionals.^105^ Referring to the band structures and DOS plots in the present situation, we again notice an increase in the band gaps in all cases within DFT-1/2 as compared to the GGA functional. Pristine bilayer *h*-BN exhibits an indirect band gap using both DFT-1/2 and GGA approximations along the Γ → *M* high-symmetry direction. The calculated values of the indirect band gap from DFT-1/2 and GGA approaches are 6.07 eV and 4.553 eV, respectively, for which an enhancement of ∼33% is observed by the former. The gap thus formed is a consequence of in-plane bonding between the p-orbitals of B and N atoms correctly described by the DFT-1/2 functional. The electronic states emerge due to the presence of H-s orbitals in the energy range of 3–4 eV (see Figure 5). We compared the band gaps obtained from DFT-1/2 and GGA functionals with the previous results from LDA/GGA^86,106^ and higher-order DFT-functionals^24,107−109^ and found them in qualitative agreement. The calculated direct band gaps of the pristine and 1, 3, 5, and 7 adsorbed hydrogen molecules from GGA and DFT-1/2 are presented in the Supporting Information The values of calculated direct band gaps from GGA and DFT-1/2 are presented in Figure 6a,b. Interestingly, by the insertion of H_2_ molecules, we notice a transition from indirect to direct band gap for both DFT-1/2 and GGA functionals (see Figure 6a–d). However, increasing the H_2_ molecular content has also reduced the band gap due to the repulsive force exerted on the B–N monolayers as discussed above (see Figure 3b).

**Figure 7:**
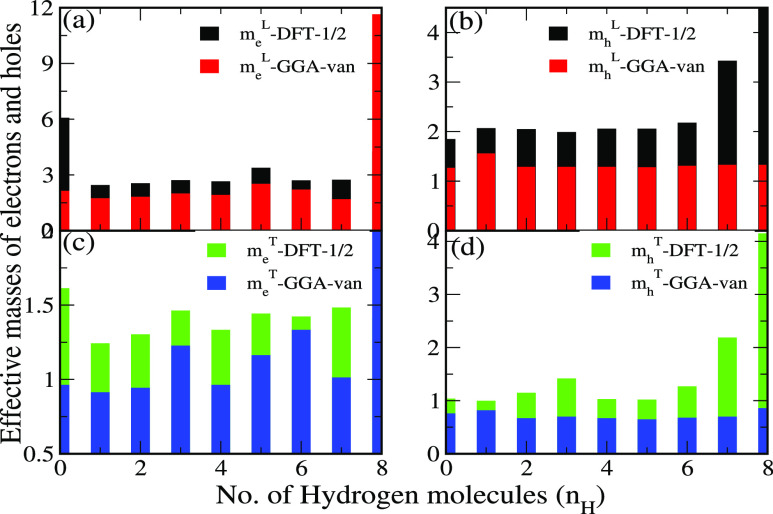
Using DFT-1/2 and GGA functionals, (a) electron effective mass along the longitudinal direction, (b) hole effective mass along the longitudinal direction, (c) electron effective mass along the transverse direction, and (d) hole effective mass along the transverse direction for bilayer *h*-BN with increasing number of *H*_2_ molecules *n*_H_ = 0, 1, 2, 3, 4, 5, 6, 7, and 8.

The effective mass of holes (*m*_h_^*^) and electrons (*m*_e_^*^) is also computed with respect to the valence band maxima and conduction band minima given by eq 4(110)
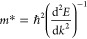
4where *E* is the band energy and *k* refers to the wave vector of the respective charge carrier. Using both DFT-1/2 and GGA functionals, effective masses along the longitudinal (∥) and transverse (⊥) directions are presented in Figure 7a,b.

For the pristine bilayer *h*-BN, effective electron mass along ∥ and ⊥ directions is 2.12 m_e_ and 0.96 m_e_, respectively, which is in reasonable agreement with the previously reported values of 2.21 (M → *L*) and 0.26 m_e_ (M → Γ).^111^ Similarly, the effective hole masses of 0.75 m_e_ (⊥) and 1.27 m_e_ (∥) are in good agreement with 0.50 m_e_ (M → Γ) and 1.33 m_e_ (M → *L*).^111^ The higher value of *m*_e_^*^ is due to the presence of a flat band along the high-symmetry M → *K* path in the conduction band region, as shown in Figure 5a. We also compute the relative electron and hole effective mass ratio (*D*) using the relation *D* = *m*_e_^*^/*m*_h_^*^ and is given in Table 1 for each case.

**Table 1:** Calculated Relative Electron and Hole Effective Mass Ratios (*D*) Using DFT-1/2 and GGA Functionals

*n*_H_	*D*_GGA_^∥^	*D*_GGA_^⊥^	*D*_DFT-1/2_^∥^	*D*_DFT-1/2_^⊥^
0	1.669	1.280	3.283	1.563
1	1.103	1.123	1.180	1.253
2	1.395	1.424	1.240	1.140
3	1.535	1.794	1.359	1.035
4	1.473	1.455	1.283	1.304
5	1.953	1.813	1.639	1.426
6	1.672	1.985	1.235	1.127
7	1.256	1.464	0.795	0.679
8	8.737	2.365	0.942	0.341

**Figure 8:**
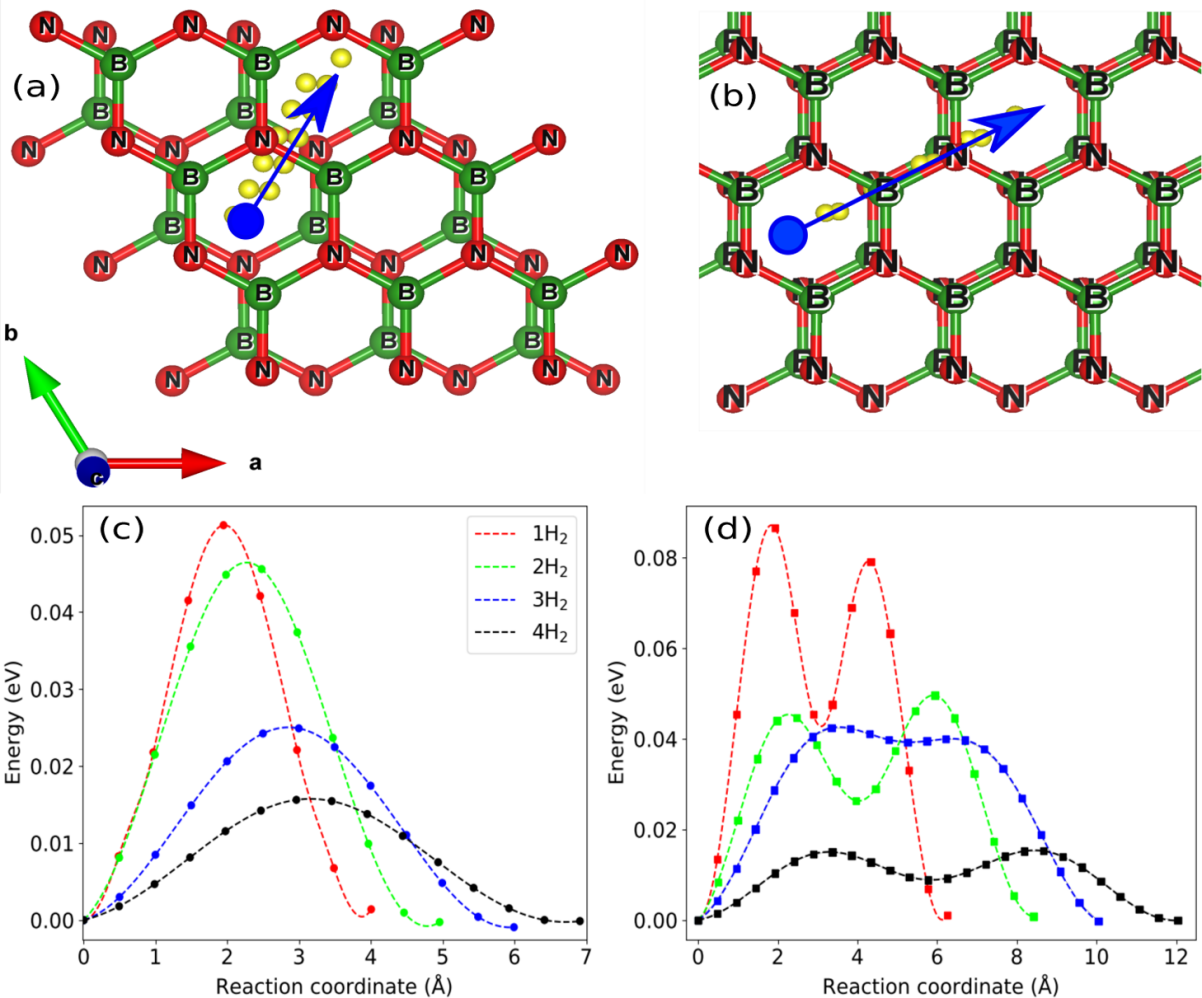
(Top) The reaction paths. (a) Bridge site and (b) bond site. (Bottom) Diffusion barriers with the reaction path alongside the (c) bridge site and (d) bond site with respect to the reaction coordinates.

Finally, we compute the H_2_ diffusion barrier taking multiple images between the initial and final ground states by means of the nudged elastic band method as shown in Figure 8. Since hollow site is the minimum-energy configuration as discussed before, two different paths are chosen for H_2_ propagation considering the bridge site (passing through the B–N bond). As can be seen in Figure 8a,b, the diffusion barrier amounts to 0.25 eV for the neighboring hexagonal site compared to 0.43 eV for the second path. For a single H_2_ with an optimized interlayer distance, the diffusion barrier amounts to 0.046 eV for the neighboring hexagonal site compared to 0.087 and 0.079 eV for the path along the bond site. Similarly, the diffusion barrier study is conducted by increasing the H_2_ molecular content in the *h*-BN bilayer cavity for both the reaction paths. We notice that the diffusion barrier decreases with an increase in the H_2_ molecular concentration for both the paths (see Figure 8c,d). The lower diffusion energy value signifies the higher hydrogen molecule adsorption and desorption capabilities (Table 2).

**Table 2:** Diffusion Barriers for Two Different Paths, that is, Through Bridge Site and via B–N Bond Site with Different H_2_ Molecular Contents

		bond site (eV)	
*n*_H_	bridge site (eV)	1st peak	2nd peak
1	0.052	0.087	0.079
2	0.046	0.044	0.049
3	0.042	0.042	0.040
4	0.024	0.015	0.015

## Conclusions

We presented theoretical insights into the possible use of bilayer *h*-BN as a potential H_2_ storage medium by means of first-principles calculations. H_2_ molecules energetically prefer the hollow site between the bilayer cavity for which structural relaxations indicate enlarged interlayer distances. With the insertion of optimal H_2_ molecular content, negative binding energies indicate stability, whereas high desorption temperatures upto six H_2_ molecules hint at a possible hindrance to reversible adsorption and desorption processes in the bilayer systems. We analyzed electronic dispersion using DFT-1/2 and GGA density functionals and found the former to correctly describe energy gaps and their nature in comparison to the latter. Moreover, effective carrier masses are calculated for each case to describe the effects of H_2_ adsorption on the electron or hole transport. Finally, diffusion barriers indicate that a small energy barrier is needed for H_2_ molecular propagation across the hexagonal minimum-energy sites. This comprehensive study forms the basis of further investigations (both theoretical and experimental) on potential H_2_-storage applications using bilayer *h*-BN cavity.

## Supporting Information Available

Convergence plot of total energy with respect to the k-mesh; electronic band structures and DOS plots of bilayer *h*-BN adsorbed with different number of H_2_ molecules; and cartesian and fractional coordinates of H_2_-adsorbed bilayer *h*-BN (PDF)
