# Supplementary material for: Hydrogen Storage in Bilayer Hexagonal Boron Nitride: A First-Principles Study
Source: ACS Omega. 2021 Nov 3;6(45):30362–70. doi: 10.1021/acsomega.1c03443 (PMC8603186; doi:10.1021/acsomega.1c03443)
Supplement: Supplementary file 1 — ao1c03443_si_001.pdf [file ao1c03443_si_001.pdf]

Supporting Information

Hydrogen Storage in Bilayer Hexagonal Boron Nitride: A First-Principles Study

Dibya Prakash Rai, *,† Bhanu Chettri, †,‡ Prasanta Kumar Patra, ‡ and Shahid Sattar *,§
†Physical Sciences Research Center (PSRC), Department of Physics, Pachhunga University College, Mizoram University, Aizawl-796001, India
‡Department of Physics, North-Eastern Hill University, Shillong, Meghalaya, 793022, India
¶Physical Sciences Research Center (PSRC), Pachhunga University College, Department of Physics, Aizawl, 796001, Mizoram, India
§Department of Physics and Electrical Engineering, Linnaeus University, SE-39231 Kalmar, Sweden
E-mail: **dibya@pucollege.edu.in; shahid.sattar@lnu.se**

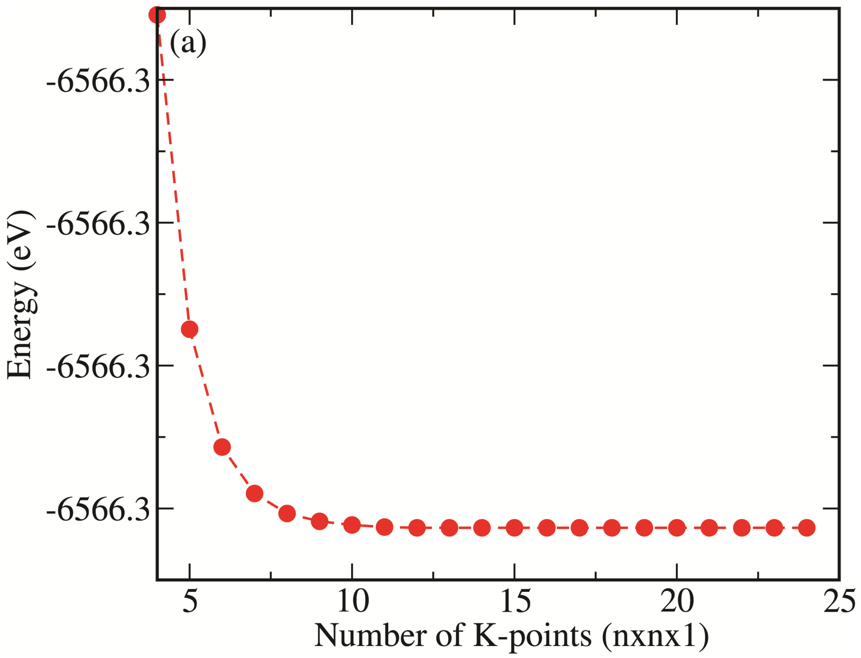

Figure S1: The convergence of total energy with respect to k-points.

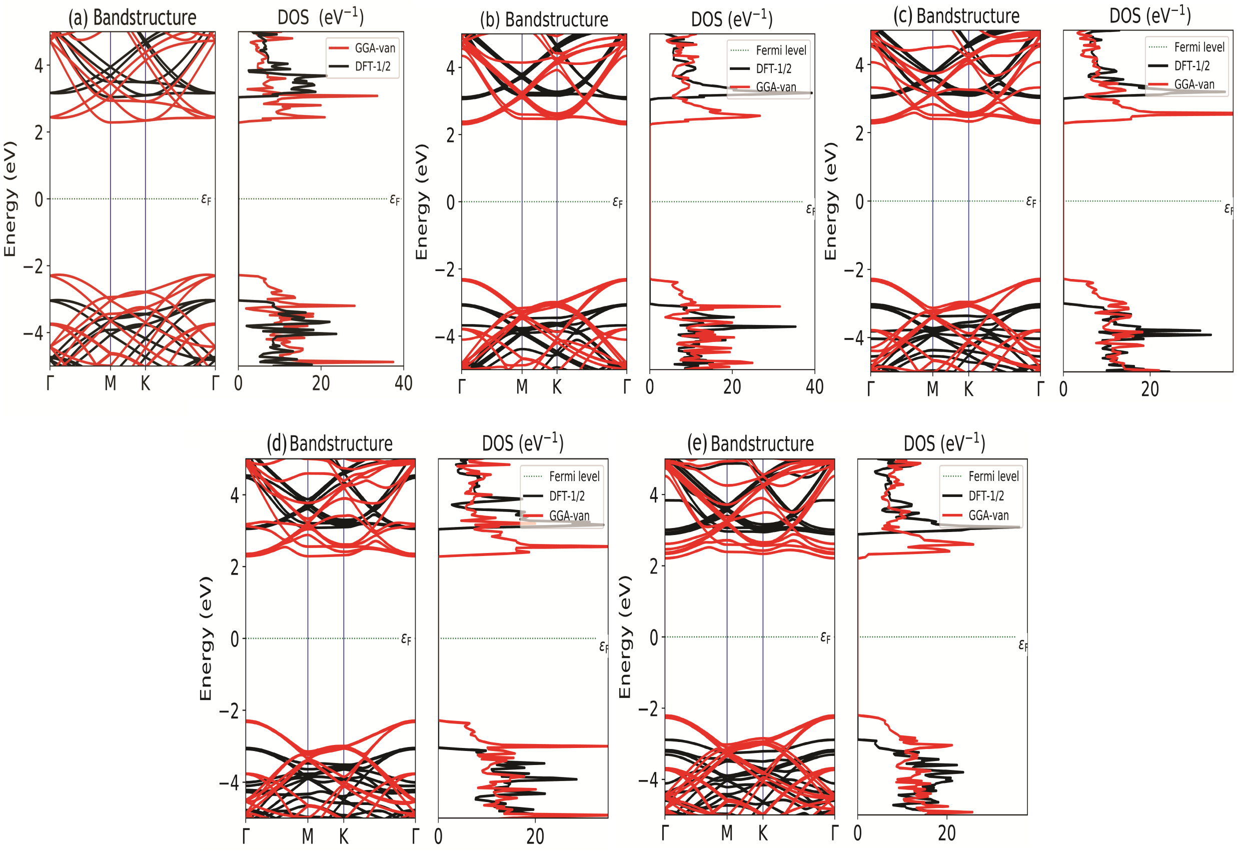

Figure S2: Electronic band structures and DOS of (a) pristine bilayer *h*-BN and (b-e) adsorbed with1, 3, 5, 7 H_2 molecules, respectively.

Cartesian and fractional coordinates of pristine bilayer *h*-BN and adsorbed with H_2 molecules.

1. h-BN bilayer

B	1.251925e+00	7.228001e-01	8.274987e+00	0.11110	0.22221	0.62113
B	2.503948e+00	2.891382e+00	8.274971e+00	0.11110	0.55555	0.62113
B	3.755985e+00	5.059958e+00	8.274987e+00	0.11110	0.88890	0.62113
B	2.503963e+00	-1.445664e+00	8.274581e+00	0.44444	0.22222	0.62110
B	3.755985e+00	7.227917e-01	8.274970e+00	0.44445	0.55555	0.62113
B	5.008022e+00	2.891382e+00	8.274971e+00	0.44445	0.88890	0.62113
B	3.755985e+00	-3.614230e+00	8.274581e+00	0.77778	0.22222	0.62110
B	5.008007e+00	-1.445664e+00	8.274581e+00	0.77778	0.55556	0.62110
B	6.260045e+00	7.228001e-01	8.274987e+00	0.77779	0.88890	0.62113
B	1.251925e+00	-7.228001e-01	5.047413e+00	0.22221	0.11110	0.37887
B	2.503963e+00	1.445664e+00	5.047819e+00	0.22222	0.44444	0.37890
B	3.755985e+00	3.614230e+00	5.047819e+00	0.22222	0.77778	0.37890
B	2.503948e+00	-2.891382e+00	5.047429e+00	0.55555	0.11110	0.37887
B	3.755985e+00	-7.227917e-01	5.047430e+00	0.55555	0.44445	0.37887
B	5.008007e+00	1.445664e+00	5.047819e+00	0.55556	0.77778	0.37890
B	3.755985e+00	-5.059958e+00	5.047413e+00	0.88890	0.11110	0.37887
B	5.008022e+00	-2.891382e+00	5.047429e+00	0.88890	0.44445	0.37887
B	6.260045e+00	-7.228001e-01	5.047413e+00	0.88890	0.77779	0.37887
N	1.252066e+00	7.228797e-01	5.046374e+00	0.11112	0.22223	0.37879
N	2.504109e+00	2.891291e+00	5.046714e+00	0.11113	0.55557	0.37881
N	3.755985e+00	5.059795e+00	5.046374e+00	0.11112	0.88888	0.37879
N	2.503960e+00	-1.445661e+00	5.046181e+00	0.44444	0.22222	0.37877
N	3.755985e+00	7.229763e-01	5.046715e+00	0.44443	0.55557	0.37881
N	5.007861e+00	2.891291e+00	5.046714e+00	0.44443	0.88887	0.37881
N	3.755985e+00	-3.614233e+00	5.046181e+00	0.77778	0.22222	0.37877
N	5.008010e+00	-1.445661e+00	5.046181e+00	0.77778	0.55556	0.37877
N	6.259904e+00	7.228797e-01	5.046374e+00	0.77777	0.88888	0.37879
N	1.252066e+00	-7.228797e-01	8.276026e+00	0.22223	0.11112	0.62121
N	2.503960e+00	1.445661e+00	8.276219e+00	0.22222	0.44444	0.62123
N	3.755985e+00	3.614233e+00	8.276219e+00	0.22222	0.77778	0.62123
N	2.504109e+00	-2.891291e+00	8.275686e+00	0.55557	0.11113	0.62119
N	3.755985e+00	-7.229763e-01	8.275685e+00	0.55557	0.44443	0.62119
N	5.008010e+00	1.445661e+00	8.276219e+00	0.55556	0.77778	0.62123
N	3.755985e+00	-5.059795e+00	8.276026e+00	0.88888	0.11112	0.62121
N	5.007861e+00	-2.891291e+00	8.275686e+00	0.88887	0.44443	0.62119
N	6.259904e+00	-7.228797e-01	8.276026e+00	0.88888	0.77777	0.62121

2. BN-1H2

B	1.233546e+00	8.334951e-01	8.874184e+00	0.10015	0.22827	0.66611
B	2.485541e+00	3.002014e+00	8.874184e+00	0.10015	0.56160	0.66611
B	3.737536e+00	5.170533e+00	8.874184e+00	0.10015	0.89494	0.66611
B	2.485541e+00	-1.335024e+00	8.874184e+00	0.43348	0.22827	0.66611
B	3.737536e+00	8.334951e-01	8.874184e+00	0.43348	0.56160	0.66611
B	4.989531e+00	3.002014e+00	8.874184e+00	0.43348	0.89494	0.66611
B	3.737536e+00	-3.503543e+00	8.874184e+00	0.76682	0.22827	0.66611
B	4.989531e+00	-1.335024e+00	8.874184e+00	0.76682	0.56160	0.66611
B	6.241526e+00	8.334951e-01	8.874184e+00	0.76682	0.89494	0.66611
B	1.270444e+00	-8.334951e-01	4.448216e+00	0.23318	0.10506	0.33389
B	2.522439e+00	1.335024e+00	4.448216e+00	0.23318	0.43840	0.33389
B	3.774434e+00	3.503543e+00	4.448216e+00	0.23318	0.77173	0.33389
B	2.522439e+00	-3.002014e+00	4.448216e+00	0.56652	0.10506	0.33389
B	3.774434e+00	-8.334951e-01	4.448216e+00	0.56652	0.43840	0.33389
B	5.026429e+00	1.335024e+00	4.448216e+00	0.56652	0.77173	0.33389
B	3.774434e+00	-5.170533e+00	4.448216e+00	0.89985	0.10506	0.33389
B	5.026429e+00	-3.002014e+00	4.448216e+00	0.89985	0.43840	0.33389
B	6.278424e+00	-8.334951e-01	4.448216e+00	0.89985	0.77173	0.33389
N	1.270444e+00	6.121842e-01	4.448216e+00	0.12207	0.21617	0.33389
N	2.522439e+00	2.780703e+00	4.448216e+00	0.12207	0.54951	0.33389
N	3.774434e+00	4.949222e+00	4.448216e+00	0.12207	0.88284	0.33389
N	2.522439e+00	-1.556335e+00	4.448216e+00	0.45541	0.21617	0.33389
N	3.774434e+00	6.121842e-01	4.448216e+00	0.45541	0.54951	0.33389
N	5.026429e+00	2.780703e+00	4.448216e+00	0.45541	0.88284	0.33389
N	3.774434e+00	-3.724854e+00	4.448216e+00	0.78874	0.21617	0.33389
N	5.026429e+00	-1.556335e+00	4.448216e+00	0.78874	0.54951	0.33389
N	6.278424e+00	6.121842e-01	4.448216e+00	0.78874	0.88284	0.33389
N	1.233546e+00	-6.121842e-01	8.874184e+00	0.21126	0.11716	0.66611
N	2.485541e+00	1.556335e+00	8.874184e+00	0.21126	0.45049	0.66611
N	3.737536e+00	3.724854e+00	8.874184e+00	0.21126	0.78383	0.66611
N	2.485541e+00	-2.780703e+00	8.874184e+00	0.54459	0.11716	0.66611
N	3.737536e+00	-6.121842e-01	8.874184e+00	0.54459	0.45049	0.66611
N	4.989531e+00	1.556335e+00	8.874184e+00	0.54459	0.78383	0.66611
N	3.737536e+00	-4.949222e+00	8.874184e+00	0.87793	0.11716	0.66611
N	4.989531e+00	-2.780703e+00	8.874184e+00	0.87793	0.45049	0.66611
N	6.241526e+00	-6.121842e-01	8.874184e+00	0.87793	0.78383	0.66611
H	2.164842e+00	1.040581e-01	6.641430e+00	0.28019	0.29618	0.49852
H	2.939903e+00	9.517475e-02	6.634052e+00	0.38405	0.39868	0.49796

3. BN-2H2

B	1.233877e+00	8.234340e-01	9.085798e+00	0.10097	0.22754	0.68199
B	2.485872e+00	2.991953e+00	9.085798e+00	0.10097	0.56088	0.68199
B	3.737867e+00	5.160472e+00	9.085798e+00	0.10097	0.89421	0.68199
B	2.485872e+00	-1.345085e+00	9.085798e+00	0.43430	0.22754	0.68199
B	3.737867e+00	8.234340e-01	9.085798e+00	0.43430	0.56088	0.68199
B	4.989862e+00	2.991953e+00	9.085798e+00	0.43430	0.89421	0.68199
B	3.737867e+00	-3.513604e+00	9.085798e+00	0.76763	0.22754	0.68199
B	4.989862e+00	-1.345085e+00	9.085798e+00	0.76763	0.56088	0.68199
B	6.241857e+00	8.234340e-01	9.085798e+00	0.76763	0.89421	0.68199
B	1.270113e+00	-8.234340e-01	4.236602e+00	0.23237	0.10579	0.31801
B	2.522108e+00	1.345085e+00	4.236602e+00	0.23237	0.43912	0.31801
B	3.774103e+00	3.513604e+00	4.236602e+00	0.23237	0.77246	0.31801
B	2.522108e+00	-2.991953e+00	4.236602e+00	0.56570	0.10579	0.31801
B	3.774103e+00	-8.234340e-01	4.236602e+00	0.56570	0.43912	0.31801
B	5.026098e+00	1.345085e+00	4.236602e+00	0.56570	0.77246	0.31801
B	3.774103e+00	-5.160472e+00	4.236602e+00	0.89903	0.10579	0.31801
B	5.026098e+00	-2.991953e+00	4.236602e+00	0.89903	0.43912	0.31801
B	6.278093e+00	-8.234340e-01	4.236602e+00	0.89903	0.77246	0.31801
N	1.270113e+00	6.222453e-01	4.236602e+00	0.12125	0.21690	0.31801
N	2.522108e+00	2.790764e+00	4.236602e+00	0.12125	0.55024	0.31801
N	3.774103e+00	4.959283e+00	4.236602e+00	0.12125	0.88357	0.31801
N	2.522108e+00	-1.546274e+00	4.236602e+00	0.45459	0.21690	0.31801
N	3.774103e+00	6.222453e-01	4.236602e+00	0.45459	0.55024	0.31801
N	5.026098e+00	2.790764e+00	4.236602e+00	0.45459	0.88357	0.31801
N	3.774103e+00	-3.714793e+00	4.236602e+00	0.78792	0.21690	0.31801
N	5.026098e+00	-1.546274e+00	4.236602e+00	0.78792	0.55024	0.31801
N	6.278093e+00	6.222453e-01	4.236602e+00	0.78792	0.88357	0.31801
N	1.233877e+00	-6.222453e-01	9.085798e+00	0.21208	0.11643	0.68199
N	2.485872e+00	1.546274e+00	9.085798e+00	0.21208	0.44976	0.68199
N	3.737867e+00	3.714793e+00	9.085798e+00	0.21208	0.78310	0.68199
N	2.485872e+00	-2.790764e+00	9.085798e+00	0.54541	0.11643	0.68199
N	3.737867e+00	-6.222453e-01	9.085798e+00	0.54541	0.44976	0.68199
N	4.989862e+00	1.546274e+00	9.085798e+00	0.54541	0.78310	0.68199
N	3.737867e+00	-4.959283e+00	9.085798e+00	0.87875	0.11643	0.68199
N	4.989862e+00	-2.790764e+00	9.085798e+00	0.87875	0.44976	0.68199
N	6.241857e+00	-6.222453e-01	9.085798e+00	0.87875	0.78310	0.68199
H	2.170300e+00	1.209476e-01	6.704248e+00	0.27962	0.29821	0.50323
H	2.945838e+00	1.132572e-01	6.707110e+00	0.38345	0.40086	0.50345
H	2.230518e+00	-2.266646e+00	6.670471e+00	0.47114	0.12272	0.50070
H	3.007898e+00	-2.262344e+00	6.674191e+00	0.57429	0.22654	0.50098

4. BN-3H2

B	1.222315e+00	8.419238e-01	9.180294e+00	0.09801	0.22742	0.68909
B	2.474310e+00	3.010443e+00	9.180294e+00	0.09801	0.56076	0.68909
B	3.726305e+00	5.178962e+00	9.180294e+00	0.09801	0.89409	0.68909
B	2.474310e+00	-1.326595e+00	9.180294e+00	0.43134	0.22742	0.68909
B	3.726305e+00	8.419238e-01	9.180294e+00	0.43134	0.56076	0.68909
B	4.978300e+00	3.010443e+00	9.180294e+00	0.43134	0.89409	0.68909
B	3.726305e+00	-3.495114e+00	9.180294e+00	0.76467	0.22742	0.68909
B	4.978300e+00	-1.326595e+00	9.180294e+00	0.76467	0.56076	0.68909
B	6.230295e+00	8.419238e-01	9.180294e+00	0.76467	0.89409	0.68909
B	1.281675e+00	-8.419238e-01	4.142106e+00	0.23533	0.10591	0.31091
B	2.533670e+00	1.326595e+00	4.142106e+00	0.23533	0.43924	0.31091
B	3.785665e+00	3.495114e+00	4.142106e+00	0.23533	0.77258	0.31091
B	2.533670e+00	-3.010443e+00	4.142106e+00	0.56866	0.10591	0.31091
B	3.785665e+00	-8.419238e-01	4.142106e+00	0.56866	0.43924	0.31091
B	5.037660e+00	1.326595e+00	4.142106e+00	0.56866	0.77258	0.31091
B	3.785665e+00	-5.178962e+00	4.142106e+00	0.90199	0.10591	0.31091
B	5.037660e+00	-3.010443e+00	4.142106e+00	0.90199	0.43924	0.31091
B	6.289655e+00	-8.419238e-01	4.142106e+00	0.90199	0.77258	0.31091
N	1.281675e+00	6.037555e-01	4.142106e+00	0.12421	0.21702	0.31091
N	2.533670e+00	2.772274e+00	4.142106e+00	0.12421	0.55035	0.31091
N	3.785665e+00	4.940793e+00	4.142106e+00	0.12421	0.88369	0.31091
N	2.533670e+00	-1.564763e+00	4.142106e+00	0.45755	0.21702	0.31091
N	3.785665e+00	6.037555e-01	4.142106e+00	0.45755	0.55035	0.31091
N	5.037660e+00	2.772274e+00	4.142106e+00	0.45755	0.88369	0.31091
N	3.785665e+00	-3.733282e+00	4.142106e+00	0.79088	0.21702	0.31091
N	5.037660e+00	-1.564763e+00	4.142106e+00	0.79088	0.55035	0.31091
N	6.289655e+00	6.037555e-01	4.142106e+00	0.79088	0.88369	0.31091
N	1.222315e+00	-6.037555e-01	9.180294e+00	0.20912	0.11631	0.68909
N	2.474310e+00	1.564763e+00	9.180294e+00	0.20912	0.44965	0.68909
N	3.726305e+00	3.733282e+00	9.180294e+00	0.20912	0.78298	0.68909
N	2.474310e+00	-2.772274e+00	9.180294e+00	0.54245	0.11631	0.68909
N	3.726305e+00	-6.037555e-01	9.180294e+00	0.54245	0.44965	0.68909
N	4.978300e+00	1.564763e+00	9.180294e+00	0.54245	0.78298	0.68909
N	3.726305e+00	-4.940793e+00	9.180294e+00	0.87579	0.11631	0.68909
N	4.978300e+00	-2.772274e+00	9.180294e+00	0.87579	0.44965	0.68909
N	6.230295e+00	-6.037555e-01	9.180294e+00	0.87579	0.78298	0.68909
H	2.167560e+00	1.337736e-01	6.696702e+00	0.27827	0.29883	0.50266
H	2.943196e+00	1.290758e-01	6.692693e+00	0.38188	0.40172	0.50236
H	2.232890e+00	-2.289323e+00	6.678325e+00	0.47320	0.12129	0.50129
H	3.008087e+00	-2.284106e+00	6.681365e+00	0.57599	0.22489	0.50151
H	2.719984e+00	3.220699e+00	6.638339e+00	0.11455	0.60962	0.49828
H	3.493762e+00	3.202275e+00	6.676897e+00	0.21897	0.71121	0.50118

5. BN-4H2

B	6.329087e-01	3.871905e-01	1.079542e+01	0.05449	0.11401	0.71969
B	1.884904e+00	2.555709e+00	1.079542e+01	0.05449	0.44735	0.71969
B	3.136899e+00	4.724228e+00	1.079542e+01	0.05449	0.78068	0.71969
B	1.884904e+00	-1.781329e+00	1.079542e+01	0.38783	0.11401	0.71969
B	3.136899e+00	3.871905e-01	1.079542e+01	0.38783	0.44735	0.71969
B	4.388894e+00	2.555709e+00	1.079542e+01	0.38783	0.78068	0.71969
B	3.136899e+00	-3.949847e+00	1.079542e+01	0.72116	0.11401	0.71969
B	4.388894e+00	-1.781329e+00	1.079542e+01	0.72116	0.44735	0.71969
B	5.640889e+00	3.871905e-01	1.079542e+01	0.72116	0.78068	0.71969
B	1.871081e+00	-3.871905e-01	3.996981e+00	0.27884	0.21932	0.26647
B	3.123076e+00	1.781329e+00	3.996981e+00	0.27884	0.55265	0.26647
B	4.375071e+00	3.949847e+00	3.996981e+00	0.27884	0.88599	0.26647
B	3.123076e+00	-2.555709e+00	3.996981e+00	0.61217	0.21932	0.26647
B	4.375071e+00	-3.871905e-01	3.996981e+00	0.61217	0.55265	0.26647
B	5.627066e+00	1.781329e+00	3.996981e+00	0.61217	0.88599	0.26647
B	4.375071e+00	-4.724228e+00	3.996981e+00	0.94551	0.21932	0.26647
B	5.627066e+00	-2.555709e+00	3.996981e+00	0.94551	0.55265	0.26647
B	6.879061e+00	-3.871905e-01	3.996981e+00	0.94551	0.88599	0.26647
N	1.871081e+00	1.058489e+00	3.996981e+00	0.16773	0.33043	0.26647
N	3.123076e+00	3.227008e+00	3.996981e+00	0.16773	0.66377	0.26647
N	4.375071e+00	5.395527e+00	3.996981e+00	0.16773	0.99710	0.26647
N	3.123076e+00	-1.110030e+00	3.996981e+00	0.50106	0.33043	0.26647
N	4.375071e+00	1.058489e+00	3.996981e+00	0.50106	0.66377	0.26647
N	5.627066e+00	3.227008e+00	3.996981e+00	0.50106	0.99710	0.26647
N	4.375071e+00	-3.278549e+00	3.996981e+00	0.83439	0.33043	0.26647
N	5.627066e+00	-1.110030e+00	3.996981e+00	0.83439	0.66377	0.26647
N	6.879061e+00	1.058489e+00	3.996981e+00	0.83439	0.99710	0.26647
N	6.329087e-01	-1.058489e+00	1.079542e+01	0.16561	0.00290	0.71969
N	1.884904e+00	1.110030e+00	1.079542e+01	0.16561	0.33623	0.71969
N	3.136899e+00	3.278549e+00	1.079542e+01	0.16561	0.66957	0.71969
N	1.884904e+00	-3.227008e+00	1.079542e+01	0.49894	0.00290	0.71969
N	3.136899e+00	-1.058489e+00	1.079542e+01	0.49894	0.33623	0.71969
N	4.388894e+00	1.110030e+00	1.079542e+01	0.49894	0.66957	0.71969
N	3.136899e+00	-5.395527e+00	1.079542e+01	0.83227	0.00290	0.71969
N	4.388894e+00	-3.227008e+00	1.079542e+01	0.83227	0.33623	0.71969
N	5.640889e+00	-1.058489e+00	1.079542e+01	0.83227	0.66957	0.71969
H	1.489355e+00	4.106782e-01	6.958443e+00	0.16670	0.22983	0.46390
H	2.158710e+00	2.089684e-02	7.003855e+00	0.28576	0.28898	0.46692
H	2.568782e+00	-2.424188e+00	6.985884e+00	0.52828	0.15564	0.46573
H	3.264502e+00	-2.765997e+00	7.022093e+00	0.64716	0.22199	0.46814
H	3.708328e+00	2.123432e+00	6.969597e+00	0.33045	0.65686	0.46464

H	4.444164e+00	2.358020e+00	6.895110e+00	0.41038	0.77284	0.45967
H	4.595167e+00	-4.856975e-01	8.216835e+00	0.64904	0.57438	0.54779
H	5.198387e+00	-3.811653e-03	8.139765e+00	0.69231	0.69172	0.54265

6. BN-5H2

B	6.329087e-01	3.871905e-01	9.325419e+00	0.05449	0.11401	0.69998
B	1.884904e+00	2.555709e+00	9.325419e+00	0.05449	0.44735	0.69998
B	3.136899e+00	4.724228e+00	9.325419e+00	0.05449	0.78068	0.69998
B	1.884904e+00	-1.781329e+00	9.325419e+00	0.38783	0.11401	0.69998
B	3.136899e+00	3.871905e-01	9.325419e+00	0.38783	0.44735	0.69998
B	4.388894e+00	2.555709e+00	9.325419e+00	0.38783	0.78068	0.69998
B	3.136899e+00	-3.949847e+00	9.325419e+00	0.72116	0.11401	0.69998
B	4.388894e+00	-1.781329e+00	9.325419e+00	0.72116	0.44735	0.69998
B	5.640889e+00	3.871905e-01	9.325419e+00	0.72116	0.78068	0.69998
B	1.871081e+00	-3.871905e-01	3.996981e+00	0.27884	0.21932	0.30002
B	3.123076e+00	1.781329e+00	3.996981e+00	0.27884	0.55265	0.30002
B	4.375071e+00	3.949847e+00	3.996981e+00	0.27884	0.88599	0.30002
B	3.123076e+00	-2.555709e+00	3.996981e+00	0.61217	0.21932	0.30002
B	4.375071e+00	-3.871905e-01	3.996981e+00	0.61217	0.55265	0.30002
B	5.627066e+00	1.781329e+00	3.996981e+00	0.61217	0.88599	0.30002
B	4.375071e+00	-4.724228e+00	3.996981e+00	0.94551	0.21932	0.30002
B	5.627066e+00	-2.555709e+00	3.996981e+00	0.94551	0.55265	0.30002
B	6.879061e+00	-3.871905e-01	3.996981e+00	0.94551	0.88599	0.30002
N	1.871081e+00	1.058489e+00	3.996981e+00	0.16773	0.33043	0.30002
N	3.123076e+00	3.227008e+00	3.996981e+00	0.16773	0.66377	0.30002
N	4.375071e+00	5.395527e+00	3.996981e+00	0.16773	0.99710	0.30002
N	3.123076e+00	-1.110030e+00	3.996981e+00	0.50106	0.33043	0.30002
N	4.375071e+00	1.058489e+00	3.996981e+00	0.50106	0.66377	0.30002
N	5.627066e+00	3.227008e+00	3.996981e+00	0.50106	0.99710	0.30002
N	4.375071e+00	-3.278549e+00	3.996981e+00	0.83439	0.33043	0.30002
N	5.627066e+00	-1.110030e+00	3.996981e+00	0.83439	0.66377	0.30002
N	6.879061e+00	1.058489e+00	3.996981e+00	0.83439	0.99710	0.30002
N	6.329087e-01	-1.058489e+00	9.325419e+00	0.16561	0.00290	0.69998
N	1.884904e+00	1.110030e+00	9.325419e+00	0.16561	0.33623	0.69998
N	3.136899e+00	3.278549e+00	9.325419e+00	0.16561	0.66957	0.69998
N	1.884904e+00	-3.227008e+00	9.325419e+00	0.49894	0.00290	0.69998
N	3.136899e+00	-1.058489e+00	9.325419e+00	0.49894	0.33623	0.69998
N	4.388894e+00	1.110030e+00	9.325419e+00	0.49894	0.66957	0.69998
N	3.136899e+00	-5.395527e+00	9.325419e+00	0.83227	0.00290	0.69998
N	4.388894e+00	-3.227008e+00	9.325419e+00	0.83227	0.33623	0.69998
N	5.640889e+00	-1.058489e+00	9.325419e+00	0.83227	0.66957	0.69998
H	1.489355e+00	4.106782e-01	6.658443e+00	0.16670	0.22983	0.49979

H	2.158710e+00	2.089684e-02	6.703855e+00	0.28576	0.28898	0.50320
H	2.568782e+00	-2.424188e+00	6.685884e+00	0.52828	0.15564	0.50185
H	3.264502e+00	-2.765997e+00	6.722093e+00	0.64716	0.22199	0.50457
H	3.708328e+00	2.123432e+00	6.669597e+00	0.33045	0.65686	0.50063
H	4.444164e+00	2.358020e+00	6.595110e+00	0.41038	0.77284	0.49504
H	4.595167e+00	-4.856975e-01	6.716835e+00	0.64904	0.57438	0.50418
H	5.198387e+00	-3.811653e-03	6.639765e+00	0.69231	0.69172	0.49839
H	3.064561e+00	4.598383e+00	6.589603e+00	0.05454	0.76138	0.49463
H	3.788899e+00	4.872266e+00	6.638464e+00	0.12991	0.87885	0.49829

7. BN-6H2

B	1.180213e+00	1.376122e+00	9.361675e+00	0.05135	0.26288	0.70270
B	2.432208e+00	3.544641e+00	9.361675e+00	0.05135	0.59621	0.70270
B	3.684203e+00	5.713160e+00	9.361675e+00	0.05135	0.92954	0.70270
B	2.432208e+00	-7.923970e-01	9.361675e+00	0.38468	0.26288	0.70270
B	3.684203e+00	1.376122e+00	9.361675e+00	0.38468	0.59621	0.70270
B	4.936198e+00	3.544641e+00	9.361675e+00	0.38468	0.92954	0.70270
B	3.684203e+00	-2.960916e+00	9.361675e+00	0.71801	0.26288	0.70270
B	4.936198e+00	-7.923970e-01	9.361675e+00	0.71801	0.59621	0.70270
B	6.188193e+00	1.376122e+00	9.361675e+00	0.71801	0.92954	0.70270
B	1.323777e+00	-1.593779e+00	3.960725e+00	0.29872	0.05373	0.29730
B	2.575772e+00	5.747404e-01	3.960725e+00	0.29872	0.38706	0.29730
B	3.827767e+00	2.743259e+00	3.960725e+00	0.29872	0.72040	0.29730
B	2.575772e+00	-3.762298e+00	3.960725e+00	0.63205	0.05373	0.29730
B	3.827767e+00	-1.593779e+00	3.960725e+00	0.63205	0.38706	0.29730
B	5.079762e+00	5.747404e-01	3.960725e+00	0.63205	0.72040	0.29730
B	3.827767e+00	-5.930816e+00	3.960725e+00	0.96538	0.05373	0.29730
B	5.079762e+00	-3.762298e+00	3.960725e+00	0.96538	0.38706	0.29730
B	6.331757e+00	-1.593779e+00	3.960725e+00	0.96538	0.72040	0.29730
N	1.323777e+00	-1.480993e-01	3.960725e+00	0.18760	0.16484	0.29730
N	2.575772e+00	2.020420e+00	3.960725e+00	0.18760	0.49817	0.29730
N	3.827767e+00	4.188939e+00	3.960725e+00	0.18760	0.83151	0.29730
N	2.575772e+00	-2.316618e+00	3.960725e+00	0.52094	0.16484	0.29730
N	3.827767e+00	-1.480993e-01	3.960725e+00	0.52094	0.49817	0.29730
N	5.079762e+00	2.020420e+00	3.960725e+00	0.52094	0.83151	0.29730
N	3.827767e+00	-4.485137e+00	3.960725e+00	0.85427	0.16484	0.29730
N	5.079762e+00	-2.316618e+00	3.960725e+00	0.85427	0.49817	0.29730
N	6.331757e+00	-1.480993e-01	3.960725e+00	0.85427	0.83151	0.29730
N	1.180213e+00	-6.955740e-02	9.361675e+00	0.16246	0.15177	0.70270
N	2.432208e+00	2.098962e+00	9.361675e+00	0.16246	0.48510	0.70270
N	3.684203e+00	4.267481e+00	9.361675e+00	0.16246	0.81843	0.70270
N	2.432208e+00	-2.238076e+00	9.361675e+00	0.49579	0.15177	0.70270

N	3.684203e+00	-6.955740e-02	9.361675e+00	0.49579	0.48510	0.70270
N	4.936198e+00	2.098962e+00	9.361675e+00	0.49579	0.81843	0.70270
N	3.684203e+00	-4.406595e+00	9.361675e+00	0.82912	0.15177	0.70270
N	4.936198e+00	-2.238076e+00	9.361675e+00	0.82912	0.48510	0.70270
N	6.188193e+00	-6.955740e-02	9.361675e+00	0.82912	0.81843	0.70270
H	1.673701e+00	1.024771e+00	6.671982e+00	0.14404	0.30157	0.50081
H	2.445676e+00	9.928820e-01	6.738999e+00	0.24926	0.40188	0.50584
H	3.152778e+00	-4.045323e+00	6.688825e+00	0.73061	0.10879	0.50207
H	3.871981e+00	-3.760200e+00	6.624279e+00	0.80444	0.22644	0.49723
H	2.329746e+00	3.516140e+00	6.615754e+00	0.03990	0.58038	0.49659
H	3.024558e+00	3.176063e+00	6.668060e+00	0.15853	0.64674	0.50051
H	3.820630e+00	5.930816e+00	6.547556e+00	0.05278	0.96443	0.49147
H	4.242854e+00	5.284835e+00	6.627997e+00	0.15863	0.97099	0.49751
H	5.204362e+00	-7.791468e-01	6.575339e+00	0.75269	0.63293	0.49356
H	4.975412e+00	-4.886460e-02	6.699965e+00	0.66609	0.65858	0.50291
H	2.650282e+00	-1.302658e+00	6.380377e+00	0.45293	0.25269	0.47892
H	3.059671e+00	-1.648928e+00	6.941102e+00	0.53404	0.28057	0.52101

8. BN-7H2

B	1.292099e+00	1.511518e+00	9.378673e+00	0.05583	0.28818	0.70398
B	2.544094e+00	3.680037e+00	9.378673e+00	0.05583	0.62151	0.70398
B	3.796089e+00	5.848556e+00	9.378673e+00	0.05583	0.95484	0.70398
B	2.544094e+00	-6.570013e-01	9.378673e+00	0.38917	0.28818	0.70398
B	3.796089e+00	1.511518e+00	9.378673e+00	0.38917	0.62151	0.70398
B	5.048084e+00	3.680037e+00	9.378673e+00	0.38917	0.95484	0.70398
B	3.796089e+00	-2.825520e+00	9.378673e+00	0.72250	0.28818	0.70398
B	5.048084e+00	-6.570013e-01	9.378673e+00	0.72250	0.62151	0.70398
B	6.300079e+00	1.511518e+00	9.378673e+00	0.72250	0.95484	0.70398
B	1.211891e+00	-1.511518e+00	3.943727e+00	0.27750	0.04516	0.29602
B	2.463886e+00	6.570013e-01	3.943727e+00	0.27750	0.37849	0.29602
B	3.715881e+00	2.825520e+00	3.943727e+00	0.27750	0.71182	0.29602
B	2.463886e+00	-3.680037e+00	3.943727e+00	0.61083	0.04516	0.29602
B	3.715881e+00	-1.511518e+00	3.943727e+00	0.61083	0.37849	0.29602
B	4.967876e+00	6.570013e-01	3.943727e+00	0.61083	0.71182	0.29602
B	3.715881e+00	-5.848556e+00	3.943727e+00	0.94417	0.04516	0.29602
B	4.967876e+00	-3.680037e+00	3.943727e+00	0.94417	0.37849	0.29602
B	6.219871e+00	-1.511518e+00	3.943727e+00	0.94417	0.71182	0.29602
N	1.211891e+00	-6.583834e-02	3.943727e+00	0.16639	0.15627	0.29602
N	2.463886e+00	2.102681e+00	3.943727e+00	0.16639	0.48960	0.29602
N	3.715881e+00	4.271200e+00	3.943727e+00	0.16639	0.82293	0.29602
N	2.463886e+00	-2.234357e+00	3.943727e+00	0.49972	0.15627	0.29602
N	3.715881e+00	-6.583834e-02	3.943727e+00	0.49972	0.48960	0.29602

N	4.967876e+00	2.102681e+00	3.943727e+00	0.49972	0.82293	0.29602
N	3.715881e+00	-4.402876e+00	3.943727e+00	0.83305	0.15627	0.29602
N	4.967876e+00	-2.234357e+00	3.943727e+00	0.83305	0.48960	0.29602
N	6.219871e+00	-6.583834e-02	3.943727e+00	0.83305	0.82293	0.29602
N	1.292099e+00	6.583834e-02	9.378673e+00	0.16695	0.17707	0.70398
N	2.544094e+00	2.234357e+00	9.378673e+00	0.16695	0.51040	0.70398
N	3.796089e+00	4.402876e+00	9.378673e+00	0.16695	0.84373	0.70398
N	2.544094e+00	-2.102681e+00	9.378673e+00	0.50028	0.17707	0.70398
N	3.796089e+00	6.583834e-02	9.378673e+00	0.50028	0.51040	0.70398
N	5.048084e+00	2.234357e+00	9.378673e+00	0.50028	0.84373	0.70398
N	3.796089e+00	-4.271200e+00	9.378673e+00	0.83361	0.17707	0.70398
N	5.048084e+00	-2.102681e+00	9.378673e+00	0.83361	0.51040	0.70398
N	6.300079e+00	6.583834e-02	9.378673e+00	0.83361	0.84373	0.70398
H	2.472928e+00	1.348169e+00	6.776896e+00	0.22558	0.43282	0.50868
H	3.218743e+00	1.244825e+00	6.589919e+00	0.33281	0.52416	0.49465
H	3.191109e+00	-3.152158e+00	6.798840e+00	0.66707	0.18254	0.51033
H	3.750891e+00	-3.498213e+00	6.388991e+00	0.76819	0.23046	0.47957
H	1.899786e+00	3.602437e+00	6.813825e+00	-0.02397	0.52977	0.51146
H	2.494141e+00	3.405720e+00	6.356558e+00	0.07027	0.59378	0.47713
H	4.102650e+00	5.011070e+00	6.654987e+00	0.16101	0.93129	0.49953
H	4.447636e+00	4.316541e+00	6.678885e+00	0.26032	0.92383	0.50133
H	4.872021e+00	-6.640314e-01	6.597836e+00	0.69960	0.59753	0.49524
H	5.638478e+00	-7.885707e-01	6.588649e+00	0.81121	0.68999	0.49455
H	2.258778e+00	-7.445915e-01	6.483257e+00	0.35792	0.24346	0.48664
H	2.875047e+00	-1.120809e+00	6.767298e+00	0.46887	0.29659	0.50796
H	5.235410e+00	1.881189e+00	6.668632e+00	0.55236	0.84153	0.50056
H	5.804141e+00	1.355642e+00	6.620733e+00	0.66846	0.87684	0.49696

9. BN-8H2

B	1.151127e+00	1.185096e+00	9.492447e+00	0.06216	0.24432	0.71252
B	2.403122e+00	3.353615e+00	9.492447e+00	0.06216	0.57766	0.71252
B	3.655117e+00	5.522134e+00	9.492447e+00	0.06216	0.91099	0.71252
B	2.403122e+00	-9.834231e-01	9.492447e+00	0.39549	0.24432	0.71252
B	3.655117e+00	1.185096e+00	9.492447e+00	0.39549	0.57766	0.71252
B	4.907112e+00	3.353615e+00	9.492447e+00	0.39549	0.91099	0.71252
B	3.655117e+00	-3.151942e+00	9.492447e+00	0.72882	0.24432	0.71252
B	4.907112e+00	-9.834231e-01	9.492447e+00	0.72882	0.57766	0.71252
B	6.159107e+00	1.185096e+00	9.492447e+00	0.72882	0.91099	0.71252
B	1.005949e+00	-1.808391e+00	4.050870e+00	0.27290	-0.00508	0.30406
B	2.257944e+00	3.601279e-01	4.050870e+00	0.27290	0.32826	0.30406
B	3.509939e+00	2.528647e+00	4.050870e+00	0.27290	0.66159	0.30406
B	2.257944e+00	-3.976910e+00	4.050870e+00	0.60623	-0.00508	0.30406

B	3.509939e+00	-1.808391e+00	4.050870e+00	0.60623	0.32826	0.30406
B	4.761934e+00	3.601279e-01	4.050870e+00	0.60623	0.66159	0.30406
B	3.509939e+00	-6.145429e+00	4.050870e+00	0.93957	-0.00508	0.30406
B	4.761934e+00	-3.976910e+00	4.050870e+00	0.93957	0.32826	0.30406
B	6.013929e+00	-1.808391e+00	4.050870e+00	0.93957	0.66159	0.30406
N	1.005949e+00	-3.627117e-01	4.050870e+00	0.16179	0.10604	0.30406
N	2.257944e+00	1.805807e+00	4.050870e+00	0.16179	0.43937	0.30406
N	3.509939e+00	3.974326e+00	4.050870e+00	0.16179	0.77270	0.30406
N	2.257944e+00	-2.531231e+00	4.050870e+00	0.49512	0.10604	0.30406
N	3.509939e+00	-3.627117e-01	4.050870e+00	0.49512	0.43937	0.30406
N	4.761934e+00	1.805807e+00	4.050870e+00	0.49512	0.77270	0.30406
N	3.509939e+00	-4.699750e+00	4.050870e+00	0.82846	0.10604	0.30406
N	4.761934e+00	-2.531231e+00	4.050870e+00	0.82846	0.43937	0.30406
N	6.013929e+00	-3.627117e-01	4.050870e+00	0.82846	0.77270	0.30406
N	1.151127e+00	-2.605835e-01	9.492447e+00	0.17327	0.13321	0.71252
N	2.403122e+00	1.907935e+00	9.492447e+00	0.17327	0.46654	0.71252
N	3.655117e+00	4.076454e+00	9.492447e+00	0.17327	0.79988	0.71252
N	2.403122e+00	-2.429102e+00	9.492447e+00	0.50660	0.13321	0.71252
N	3.655117e+00	-2.605835e-01	9.492447e+00	0.50660	0.46654	0.71252
N	4.907112e+00	1.907935e+00	9.492447e+00	0.50660	0.79988	0.71252
N	3.655117e+00	-4.597621e+00	9.492447e+00	0.83993	0.13321	0.71252
N	4.907112e+00	-2.429102e+00	9.492447e+00	0.83993	0.46654	0.71252
N	6.159107e+00	-2.605835e-01	9.492447e+00	0.83993	0.79988	0.71252
H	1.937657e+00	9.817261e-01	6.799628e+00	0.18249	0.33340	0.51039
H	2.699355e+00	8.318002e-01	6.786010e+00	0.29541	0.42327	0.50937
H	3.273634e+00	-3.337815e+00	6.856968e+00	0.69232	0.17925	0.51469
H	3.601140e+00	-3.920936e+00	6.464576e+00	0.78074	0.17803	0.48524
H	1.876874e+00	3.387163e+00	6.955077e+00	-0.01048	0.51018	0.52206
H	2.281284e+00	3.039828e+00	6.392072e+00	0.07005	0.53732	0.47980
H	4.148709e+00	3.672341e+00	6.932100e+00	0.27003	0.83453	0.52033
H	4.686138e+00	3.207307e+00	6.623714e+00	0.37732	0.87033	0.49719
H	4.015996e+00	-1.219459e+00	6.647890e+00	0.62834	0.44089	0.49900
H	4.802282e+00	-1.101235e+00	6.651755e+00	0.72392	0.55465	0.49929
H	1.654437e+00	-1.040493e+00	6.656845e+00	0.30021	0.14027	0.49967
H	2.320574e+00	-1.407728e+00	6.812198e+00	0.41711	0.20072	0.51133
H	4.834167e+00	1.180967e+00	6.836356e+00	0.55276	0.73429	0.51315
H	5.593909e+00	1.274782e+00	6.714011e+00	0.64669	0.84264	0.50396
H	6.053201e+00	-8.682637e-01	6.753157e+00	0.87254	0.73908	0.50690
H	6.839349e+00	-7.482096e-01	6.756953e+00	0.96797	0.85295	0.50719
